# "A Promise Unfulfilled": Stakeholder Influence and the 2018 UN High-Level Meeting on NCDs: Comment on "Competing Frames in Global Health Governance: An Analysis of Stakeholder Influence on the Political Declaration on Non-communicable Diseases"

**DOI:** 10.34172/ijhpm.2021.140

**Published:** 2021-10-03

**Authors:** Johanna Ralston

**Affiliations:** World Obesity Federation, London, UK.

**Keywords:** Industry, Non-communicable Diseases, NCD Targets, Multisectoral, High-Level Meeting, Lived Experience

## Abstract

In recognition of the global burden of non-communicable diseases (NCDs), the past decade has seen three United Nations High-Level Meetings (UN HLMs) on NCDs. Yet progress in terms of political or financial commitments has been very slow. At the 2018 meeting, a political declaration was approved but featured language that had been watered down in terms of commitments. In "Competing Frames of Global Health Governance: An Analysis of Stakeholder Influence on the Political Declaration on Non-communicable Diseases," Suzuki et al analyze the documents that were submitted by Member States, non-governmental organizations and the private sector during the consultation period and conclude that the private sector and several high-income countries (HICs) appeared to oppose regulatory frameworks for products associated with NCDs, that wealthier countries resisted financing commitments, and that general power asymmetries affected the final document. This comment supports their findings and provides additional considerations for why the NCD response has yet to produce significant commitments.

 In late 2017 United Nations (UN) Secretary-General Antonio Guterres issued a report as follow up to the Millennium Summit, “Progress on the Prevention and Control of Non-communicable Diseases.” The report was clear that progress was inadequate, and that failures to meet commitments were a promise unfulfilled.^1^ The following September the Third High-Level Meeting (HLM) on non-communicable diseases (NCDs) took place at the time of the UN General Assembly. This would be the first UN and World Health Organization (WHO) meeting on the topic under Director General Tedros Gebreyesus, who had just appointed a High-Level Commission on NCDs co-chaired by the Presidents of Finland, Uruguay and Sri Lanka, the Minister of Health of Russia, and Pakistani Special Assistant to the Prime Minister Sania Nishtar. Yet the 2018 meeting continued a pattern that had started with earlier HLMs, of significant resources and plans failing to translate into a sufficiently ambitious resolution. This result is thoughtfully analyzed by Mao Suzuki of University of Southern California and Douglas Webb and Roy Small of the United Nations Development Programme, “Competing Frames in Global Health Governance: An Analysis of Stakeholder Influence on the Political Declaration on Non-communicable Diseases.”^2^

 Their paper is an important analysis of what has long been anecdotally shared, of how some stakeholders from the private sector appear to have influenced changes in the political declaration and its concrete commitments, either by direct pressure or indirect action at the Member State level. As the authors note, this has likely weakened the general NCD response. At the same time, assumptions about the primacy of private sector interference in the process require further testing and analysis, and there are clearly other factors that have contributed to a process in which commitments fell short of expectations, including from Member States representing high-income countries (HICs). This comment will explore in more detail this assertion in relation to framing, language, representation, and power asymmetry.

 The study methodology is based on detailed review of the many documents submitted as part of the original drafting of the Political Declaration and the subsequent consultation processes. Outlining a taxonomy of policy positions advocated by various stakeholders in the development of the Declaration, the authors document and analyze changes in the text between the original draft and the final version, and link to which language was championed by which actor. From this they draw conclusions about which positions and which champions for that position were influential.

 At the outset the authors describe the process as one of competing frames. The debate about the role framing plays with respect to the NCD agenda has been raised in several ways in recent years, partly in response to continued frustration that HLMs and the resulting political commitments have not led to more resources. The relative lack of concerted action on NCDs has variously been framed in terms of political economy, commercial and social determinants, neoliberalism,^3^ and social justice, to mention a few, without consensus on a satisfactory common definition other than that there are multiple frames, and they contradict one another.

 There are several ways in the process leading up to the HLM in which misleading language played a role. For example, the world “consultation” implies an unbiased and benign process of consideration for all views, yet the supposedly even playing field appears to have enabled inappropriate influence by more powerful stakeholders. Many non-governmental organizations suggested that words such as “voluntary” were intentionally introduced by industry to weaken commitments in the Declaration, while language around regulation (taxation) of tobacco, alcohol and unhealthy food appeared in over 50 submitted statements, yet the word tax was omitted from the final Declaration. In these cases, language has contributed to impeding action, further illustrated by the inclusion of commercial entities under the broad umbrella of “non-state actors,” implying the same motivations as civil society on a particular topic.

 Yet language also suggests a more adversarial role between and among sectors than may necessarily be the case. For example, the term “health-harming industry” can be helpful in enumerating that discrete yet significant elements in the private sector contribute to ill health and mortality through products that damage people and the planet. However, the reactive clustering of all private sector under one umbrella term “industry” misses out on how diverse the food sector is, for example, and blocks potentially positive and innovative solutions. Another misleading term, often supported by HIC and private sector submissions, is “healthy lifestyles,” which suggests marketing more than policy, and implies a level of individual agency that is often not present.

 The power imbalance in the process leading up to and during the 2018 HLM clearly contributed to a lack of robust commitments and is mirrored in the number of heads-of-government who attended the meeting itself: only 23, despite presumably all heads of state and government being in New York for the UN General Assembly. While the meeting succeeded in extending the four-by-four diseases and risk factors to a five-by-five framework,^4^ it was also meant to once and for all address the gap between rhetoric and realistic action by addressing chronic challenges including lack of donor funding, insufficient political will and national capacity, lack of coherence and tradeoffs in policies, some criticisms of the best buys, and industry interference. While there is need to reform the consultative process to dilute the influence of private sector, the wider issue of ensuring that representation by other sectors be strengthened is not addressed.

 The other question is why the global response between the first meeting in 2011 and the third one in 2018 had not been greater when the case had already been made so effectively. In the years leading up to the original HLM on NCDs in 2011, three publications in particular were galvanizing in the evidence they put forward about the urgency of the NCD pandemic. The first, the Port of Spain Declaration, was the output of a meeting convened by Caribbean heads of state and health actors to call for concerted action on NCDs which were clearly supplanting infectious disease on most of the islands. The next, “Where Have All the Donors Gone?”^5^ by Rachel Nugent and Andrea Feigl, confirmed an overwhelming imbalance between NCD mortality and overall funding for health. Their conclusions - that less than three percent of development assistance for health has been allocated to NCDs - have been reinforced repeatedly in the decade since their study was published: numbers have hovered between two and four percent in recent years, while NCDs now drive around 70% of all deaths.^6^ The third breakthrough publication was the World Economic Forum annual risk report for 2010, which listed global chronic diseases as third most serious risk in terms of severity and likelihood, ahead of other potentially cataclysmic events including extreme weather, pandemics, and international terrorism.^7^

 The combined evidence and influence of these reports had the potential to generate a huge amount of resource and attention to NCDs, yet this did not happen. Often compared to the level of political and financial support that was mobilized following the UN General Assembly Special Session on AIDS in 2001, the three HLMS on NCDS since 2011 have fallen well below expectations and hopes. In analyzing the drivers of this, Suzuki et al note that the most critical one has been the inherent incompatibility between free trade policies in tobacco and food driving diseases that health policies are aiming to reduce. They also suggest that while financing commitments were mentioned in 22 separate contributions to the 2018 declaration, a continued aversion among HICs led to no concrete commitments whatsoever. The authors further restate that the process of consultation itself, giving equal footing to well-funded and shareholder-beholden industry and high-income country governments versus civil society and low- and middle-income country governments contributed to the weak commitments, whether due to “neoliberal ideological assumptions or calculations of political costs.”

 But what else might have contributed to what the authors describe as “charges of inadequacy” in NCD responses? Some further challenges are listed below:

1. The absence of a political leader playing the role the late UN SG Kofi Annan played in AIDS in pushing through commitments made in the original political declaration, alongside significant support from donors including Bill Gates.
2. The inherent complexity of at least four diseases and four risk factors combined in a single agenda, exacerbated by the awkwardness of the term non-communicable with its negative prefix suggesting non-urgency. This is further complicated by the term “multisectoral,” which is vital but not resonant with a wider public. There are also inherent challenges of aligning, incentivizing and holding accountable different sectors: diseases with single vectors and fewer and less complex drivers can be addressed mainly in the health sector, but while NCDs share many risk factors, they do not lend themselves to simple single solo solutions.
3. Absence of a strong patient voice. In a movement that had its origins in professional societies and WHO, the original omission of patients as core stakeholders was a pragmatic matter of prioritization. At the outset of the NCD movement, the goal was to achieve coordination across global and national health departments and among professional federations that were focused on the main diseases and risk factors. This meant the galvanizing nature of activist populations who have been directly affected by a disease was largely missing in the years leading up to the 2018 HLM. Also missing has been recognition of their unique expertise in navigating and managing NCDs, which require interactions with multiple systems, ideally with the affected individual at the center from the beginning.

It is increasingly argued that the very nature of global health is asymmetrical and rooted in colonial structures and mindsets, with financing from high income country donors going to high income country-headquartered institutions to carry out work, led by HIC experts, that affects LMICs. It has been noted that HICs account for a “majority of global health spending, and by controlling the purse strings, they effectively control the global health agenda.”^8^ Thus, efforts to simply remove industry interference should be taken in a larger context of power, including that held by established health donors.

 In the end, the authors’ assertion that private sector responses to various iterations of the Political Declaration diluted commitments is convincing, though the process itself is so iterative that it seems hard to assign a single outcome to a single driver. Indeed, the complexity of NCDs has been compared to the complexity of climate change^9^; how do we effectively communicate the urgency and existential threat of both in ways that generate action, overcome commercial interests and lack of coordination, and distribute action and accountability equitably? And how do we change the incentives and frameworks that keep shareholders from demanding and companies from producing healthier products?

 Like climate change, the most effective sources of change may come from changing the consumer paradigm and from youth. One of the more startling indictments of sugar sweetened beverages, in many ways a proxy for wider concerns about our distorted incentives in food systems, came during a recent press conference with Cristiano Ronaldo, whose popularity and influence are evident in over 350 million Instagram followers. At the start of the conference, he shoved aside the bottle of soda that had been placed before him and said, “aqua.” Though some claimed it was just a coincidence, the sponsoring company reportedly lost $4 billion in market value the next day.

## Acknowledgements

 Thank you to colleagues at the World Obesity Federation for exchanging ideas on these issues and to NCD leaders including Sania Nishtar, Sir George Alleyne and Sir Trevor Hassell.

## Ethical issues

 Not applicable.

## Competing interests

 Author declares that she has no competing interests.

## Author’s contribution

 JR is the single author of the paper.
